# Favorable Response to Mirtazapine in John Cunningham Virus-related Gray Matter Lesion in a Patient with Human Immunodeficiency Virus

**DOI:** 10.7759/cureus.4255

**Published:** 2019-03-14

**Authors:** Abdullah I Alwehaibi, Mohammed I AlJaber, Shahpar Nahrir

**Affiliations:** 1 Surgery, King Saud Medical City, Riyadh, SAU; 2 Miscellaneous, Al-Imam Mohammad Ibn Saud Islamic University, Riyadh, SAU; 3 Neurology, King Saud Medical City, Riyadh, SAU

**Keywords:** pml, mirtazapine, gray matter, hiv/aids, jcv, favorable response, arab

## Abstract

Mirtazapine has recently emerged as a promising agent for the treatment of progressive multifocal leukoencephalopathy (PML). While there is no Class I evidence for its use, numerous case reports have illustrated mirtazapine’s efficacy. True to its name, PML is known to occur mostly in the white matter of the brain as its causative agent, John Cunningham virus (JC virus), has a predilection for infecting glial cells. The virus replicates vigorously in oligodendrocytes and causes lysis of the glial cell culminating in demyelination. Therefore, gray matter involvement is rare. Mirtazapine’s 5HT2A receptor blocking capacity is presumed to hinder JC virus’ entry into glial cells. We report a case of a patient with human immunodeficiency virus (HIV) with predominantly gray matter lesions from JC virus reactivation. This case is the first reported case of gray matter PML in an Arabic patient who responded favorably to mirtazapine therapy.

## Introduction

John Cunningham virus (JC virus) is notorious for being highly lethal. Non-archetype JC virus is harmful to brain cells. Archetypal JC virus is ubiquitous in distribution; its worldwide seroprevalence is approximately 80% [1]. JC virus deoxyribonucleic acid (DNA) has been detected in oligodendrocytes, astrocytes, lymphocytes, kidney epithelium cells, tonsil stromal cells, and plasma cells of healthy individuals and is periodically shed in urine [2-3].

The virulence of JC virus is established when extensive deletions and duplications in the nucleotide sequences within the promoter/enhancer region of the genome occur [4]. The resultant pathogenic variant acquires a tandem repeat of a 98-bp element (various Mad strains) [5]. This process is triggered by a loss of protective cellular immunosurveillance. This pathogenic variant, upon entry into glial cells, causes extensive myelin destruction. As it commonly affects the oligodendrocytes, lesions are seen more frequently in the white matter. However, gray matter is also composed of (to a lesser extent) some glial cells and myelinated fibers [5]. Therefore, JC virus-related deep gray matter lesions are not impossible.

Nevertheless, wherever it affects in the brain, JC virus leads to disastrous consequences. In the era before highly active antiretroviral therapy, the prognosis for patients with progressive multifocal leukoencephalopathy (PML) was grave; death occurred in approximately 95% of patients within four to six months of diagnosis [6]. Those who survive PML can be left with severe neurological disabilities. Despite the dire prognosis, there is no specific established treatment modality for PML. There is, however, is an urge in the scientific community to explore different agents to counteract the deleterious effects of JC virus. One such endeavor is the use of the 5HT2A receptor blocker mirtazapine, and the successful use of mirtazapine has been reported in several case reports. We present a case where a male patient of Arab ancestry diagnosed with acquired immunodeficiency syndrome (AIDS) was found to have basal ganglion and thalamic lesions who responded remarkably to mirtazapine therapy.

## Case presentation

A 37-year-old Yemeni male patient was brought to the hospital by his coworker after the patient had been disoriented for several days. A report from another medical center indicated the patient was diagnosed with tuberculosis (TB). According to this report, he had presented to the other facility one month previous with fever, productive cough, and disorientation. He was advised to start anti-TB therapy (ATT). However, the lack of collateral history made it impossible to confirm whether he had been receiving ATT. At presentation to our center, he was drowsy but arousable to strong verbal commands. He uttered a few incomprehensible sounds, and he obeyed spoken commands only occasionally. He appeared to have mild weakness in his left arm. His cranial nerve examination showed no abnormalities. The results of his systemic examination were unremarkable: we noted no skin rash, indications of intravenous drug use, evidence of peripheral stigmata of infective endocarditis, or lymphadenopathy. On subsequent days, he sustained several attacks of generalized tonic-clonic seizures. Given that he was febrile with evidence of meningismus, he underwent lumbar tap.

Investigations

**Figure 1:**
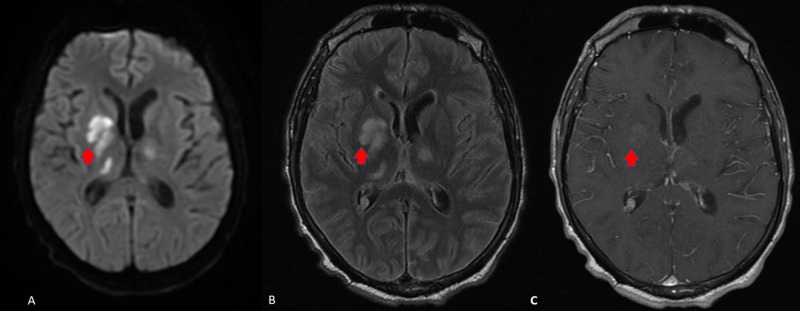
Brain magnetic resonance imaging (MRI) at presentation, prior to treatment with mirtazapine A) diffusion-weighted imaging (DWI); B) fluid-attenuated inversion recovery (FLAIR); C) contrast study. Lesion visible over deep gray matter of basal ganglion and thalami.

His cerebrospinal fluid (CSF) was light yellowish with 373 cells (73% monomorphic) with glucose at 2.4 g/dL, protein at 620 g/dL. The CSF was positive for JC virus DNA with a viral load of 2800 copies/mL. His serology for human immunodeficiency virus (HIV) was positive with a CD4 count of 135 /µL and an HIV viral load of 179,795 copies/mL. Test results for hepatitis B virus and sputum acid-fast bacilli were negative. CSF polymerase chain reaction results for TB were negative, so was the culture for TB in the CSF sample. However, the report from the peripheral center had shown positive TB culture in CSF. Computed tomography (CT) of the patient’s brain revealed left basal ganglia hypodensity and left periventricular white matter hypodensity. Magnetic resonance imaging (MRI) showed bilaterally scattered patchy confluent subcortical and periventricular foci confirmed on T2-weighted images (T2WI) and fluid-attenuated inversion recovery (FLAIR) images (Figure 1). We noted diffusion-weighted imaging (DWI) restriction at the left frontal lobe, bilateral basal ganglia as well as in the brainstem and cerebellar vermis. The largest area of DWI restriction was seen over the right basal ganglia lesion which also showed faint heterogeneous enhancement in the post-contrast images with mild mass effect on the adjacent right lateral ventricle.

Treatment

Based on clinical, imaging, and virological finding, we decided to treat the patient with combined antiretroviral therapy (cART) along with the ATT. Given the reported cases of successful treatment with an anti-5HT2A antagonist, we treated the patient with mirtazapine along with standard cART and ATT.

Outcome

**Figure 2:**
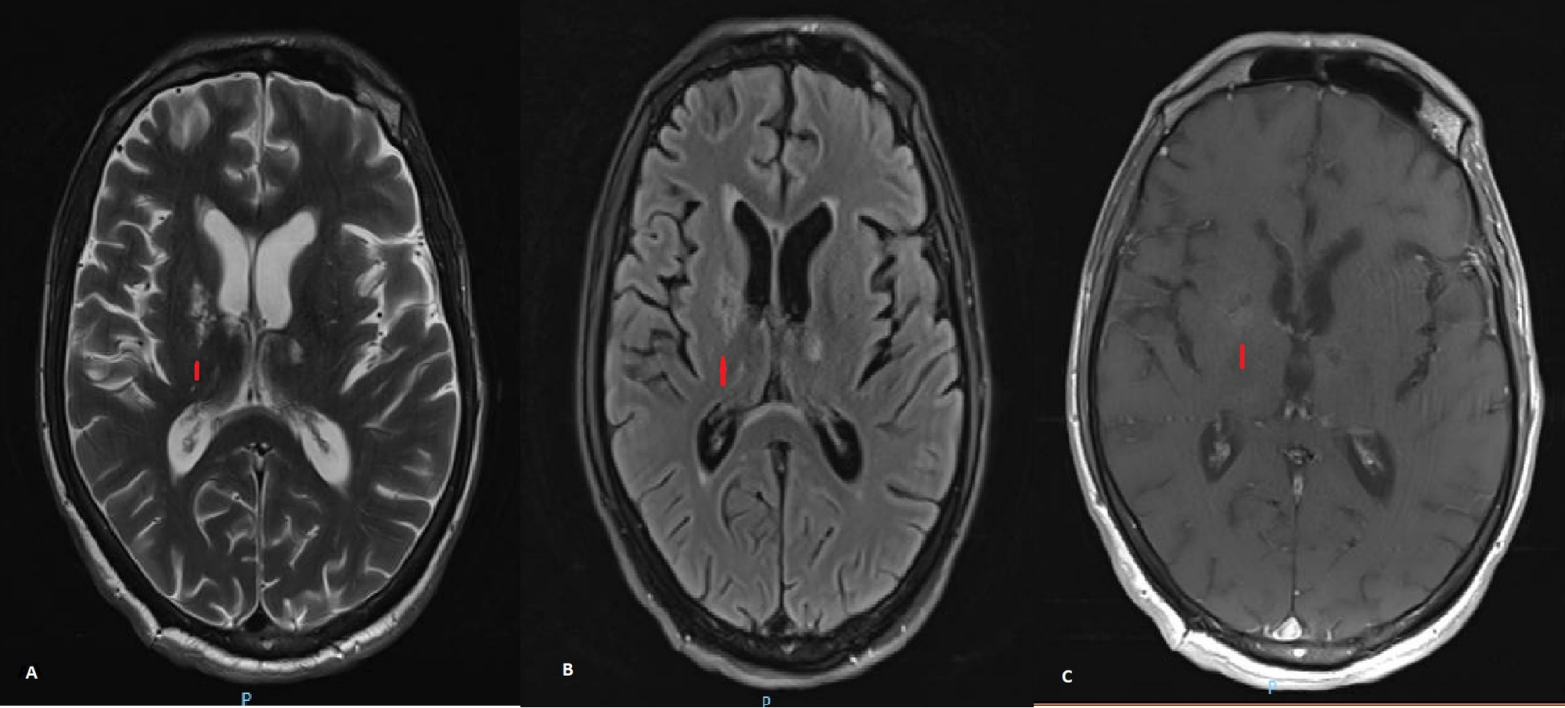
Brain magnetic resonance imaging (MRI) six weeks after mirtazapine therapy A) T2-weighted images (T2WI) showing marked resolution of previously seen lesion over gray matter; B) fluid-attenuated inversion recovery (FLAIR) image demonstrating similar resolution of lesion size; C) contrast study shows no discernible area of contrast uptake.

The patient remained clinically stable with no apparent worsening of his neurological deficit. He became more alert and could reply to one or two questions coherently. He sustained no further seizures. He developed rigidity in all limbs and bradyphrenia. He also became hypophonic and apathetic. A second MRI after six weeks of therapy revealed remarkable improvement (Figure 2). After six months of treatment, the patient is more alert, obeys commands, and can communicate with two to three sentences at a time. His weakness has resolved, but he has residual rigidity over his limbs.

## Discussion

PML is a demyelinating disease caused by JC virus, a polyomavirus that affects CNS white matter. This disease was first noted by Astrom et al. in 1958 based on the characteristic histopathological features of demyelination, bizarre astrocytes, and enlarged oligodendroglia nuclei [7]. JC virus was later isolated from the brain of a patient named John Cunningham in 1971. JC virus vigorously replicates in glial cells and results in extensive myelin breakdown and white matter destruction. The virus is thought to act on the 5HT2A receptor on the glial cells to gain entry into the cell [8]. 5HT2 receptors are expressed in the brain microvasculature and on astrocytes at the blood-brain barrier [9]. In the pre-HIV era, PML was infrequent and occurred mostly in patients who were immunocompromised from underlying hematologic malignancies or immunosuppressive medications, or transplant recipients and patients of advanced age [7]. In the last four decades, PML has occurred in greater frequency in AIDS patients compared with patients in an immunosuppressed state from other conditions [10]. The first description of PML in an AIDS patient was reported in 1982 [11]. The estimated risk of developing PML in AIDS patients range from 1% to 4% [12]. A study in the U.S. showed that 82% of the 9,675 PML cases documented from 1998 to 2005 were associated with HIV infections [13]. PML is known to occur in both HIV-1 and HIV-2 infected patients. Akin to our patient, most HIV-infected patients develop PML as they approach a poor immunological status expressed by a low CD4 cell count (< 200/µL) [14-15]. The fundamental pathological feature of PML is demyelination [16]. Demyelination typically occurs as a multifocal process; it can occur in any location in the white matter and range in size from 1 mm to several centimeters [16]. A JC virus infection in gray matter structures such as the basal ganglia or thalamus is a rare occurrence. Theoretically, a JC virus infection in gray matter is plausible as gray matter also possess some degree of myelinated fiber and glial cells. Lesions of the deep gray structures were noted in a study of 47 HIV patients with PML, and basal ganglia lesions were found in 7% of the patient and thalamic lesions were found in 8% [17]. The common clinical findings are behavioral and cognitive abnormalities which are seen in one-third to one-half of all patients. Motor weakness, gait abnormalities, visual field deficits, speech and language disturbances, and incoordination are also seen in PML [16]. Sensory loss, seizures, headache, and diplopia occur less frequently [16]. In neuroimaging, MRI shows hyperintense lesions on T2WI and FLAIR images in the affected regions [16]. Classically, PML lesions do not show edema, mass effect or contrast enhancement on imaging [5]. The resultant infection generally remains refractory to treatment.

There is currently no specific treatment available for this catastrophic illness. However, the pragmatic approach targets the basic mechanism of action of the virus. This includes immune response modulator agents that inhibit JC virus entry into the glial cells and inhibit JC virus DNA replication [18]. Combined antiretroviral therapy is considered the first-line of therapy as they are believed to counteract JC virus DNA replication and aid in the restoration of the adaptive immune response to JC virus. However, a plethora of other agents with different indications have surfaced recently as promising JC virus glial cell entry inhibitors. Mirtazapine is one of these reported drugs with demonstrated success in a small series of HIV-positive PML cases [19-20]. Mirtazapine is an alpha-2-adrenergic, 5HT2A- and 5HT3-receptor antagonist that can cross the blood-brain barrier [19]. By limiting JC virus entry into the glial cells, mirtazapine may retard the adverse effect of its infection. While the mechanism is promising, a study published in 2016 systemically reviewed data from 2005 to 2015 of five cohort studies and 74 case reports found only the natalizumab-induced PML cases responded positively to mirtazapine treatment [19].

Our case presents a favorable response to mirtazapine in an HIV-related, predominantly gray matter affected PML. Nevertheless, as this case had quite a few atypical features without a histopathological confirmation of PML therefore, we can not completely rule out having other less likely differentials for the lesions seen in MRI brain and for the rapid response to combination treatment (cART, Mirtazapine, and ATT). In other words, it may not be incorrect to presume that there was concomitant CNS TB and restoration of ATT use may have influenced the quick resolution of the brain lesions. However, as CSF TB culture was negative at our center, we were hesitant to make the diagnosis of TB. Instead, in our patient, JC virus DNA was detected by PCR in the CSF, the presence of which is generally considered highly specific for the diagnosis of PML. Therefore, we feel it was most likely a case of predominantly gray matter involved PML in an HIV positive patient.

## Conclusions

This case represents the first report of PML in the gray matter of an Arab patient with HIV who responded well to mirtazapine treatment in conjuction with cART.
